# Prognostic significance of lung radiation dose in patients with esophageal cancer treated with neoadjuvant chemoradiotherapy

**DOI:** 10.1186/s13014-019-1283-3

**Published:** 2019-05-24

**Authors:** Jhen-Bin Lin, Li-Chung Hung, Ching-Yuan Cheng, Yu-An Chien, Chou-Hsien Lee, Chia-Chun Huang, Tsai-Wei Chou, Ming-Huei Ko, Yuan-Chun Lai, Mu-Tai Liu, Tung-Hao Chang, Jie Lee, Yu-Jen Chen

**Affiliations:** 1 Department of Radiation Oncology, Changhua Christian Hospital, 135 Nanhsiao Street, Changhua City, 50006 Taiwan; 2 Department of Thoracic Surgery, Changhua Christian Hospital, Changhua, Taiwan; 3 Department of Radiation Oncology, Changhua Christian Hospital Yunlin Branch, Yunlin, Taiwan; 4 Department of Radiation Oncology, E-Da Cancer Hospital, Kaohsiung, Taiwan; 5 Division of Medical Physics, Department of Radiation Oncology, Changhua Christian Hospital, Changhua, Taiwan; 6 Department of Medical Imaging and Radiological Technology, Yuanpei University of Science and Technology, Hsinchu, Taiwan; 7 Department of Radiation Oncology, MacKay Memorial Hospital, 92, Section 2, Chung Shan North Road, Taipei, 10449 Taiwan; 8 Department of Medicine, MacKay Medical College, New Taipei city, Taiwan

## Abstract

**Background:**

The prognostic significance of radiation dose to the lung or heart is unknown in esophageal cancer patients receiving neoadjuvant chemoradiotherapy followed by surgery (trimodal therapy). This study aimed to determine the association between lung and heart radiation dose volumes and prognosis of esophageal cancer after trimodal therapy.

**Methods:**

This study reviewed 123 esophageal cancer patients treated with trimodal therapy in two tertiary institutions between 2010 and 2015. The dose-volume histogram parameter of Vx was defined as the percentage of total organ volume receiving a radiation dose of x (Gy) or more. Predictors of overall survival (OS) were identified using Cox regression models. Receiver-operating characteristic curves were used to select cut-off values for dose-volume.

**Results:**

Median follow-up was 28.3 months (range: 4.7–92.8 months). Median OS and progression-free survival were 34.0 months (95% confidence interval [CI]: 27.4–40.6 months) and 24.8 months (95% CI, 18.9–30.7 months), respectively. Multivariate analyses showed that lung V20 (hazard ratio, 1.09; 95% CI: 1.04–1.14; *p* < 0.001) and lung V5 (hazard ratio, 1.02; 95% CI: 1.00–1.05; *p* = 0.03) were associated with OS when adjusting for surgical margin and pathological treatment response. The 5-year OS for patients with lung V20 ≤ 23% vs. patients with lung V20 > 23% was 54.4% vs. 5% (*p* < 0.001) whereas that for patients with lung V5 ≤ 56% vs. patients with lung V5 > 56% was 81.5% vs. 23.4% (*p* < 0.001). Mean heart dose showed no association with survival outcomes.

**Conclusions:**

Lung radiation dose was independently associated with survival outcomes in esophageal cancer patients treated with neoadjuvant chemoradiotherapy and surgery.

**Electronic supplementary material:**

The online version of this article (10.1186/s13014-019-1283-3) contains supplementary material, which is available to authorized users.

## Introduction

Esophageal cancer is an aggressive and lethal malignancy, with 455,800 new cases and 400,200 deaths occurring annually worldwide [1]. However, the prognosis of esophageal cancer treated with curative surgery alone is relatively poor, and multimodal treatments have been developed to improve survival [2–5]. Neoadjuvant chemoradiotherapy (NCRT) has been suggested for improvement of complete resection rate and survival rate. The CROSS trial confirmed the benefit of NCRT among esophageal cancer patients amenable to surgery [6, 7]; hence, NCRT followed by surgery (trimodal therapy) has become the mainstay of treatment for esophageal cancer [6–11].

The lungs and the heart are the two main organs at risk in thoracic radiotherapy. Previous studies have shown the detrimental effects of higher lung or heart radiation dose-volumes in lung cancer radiotherapy [12–18]. However, the radiotherapy protocol in esophageal cancer is different from that in lung cancer in terms of central location of target volume, wherein it may contribute to high radiation dose-volume of lung or heart (Additional file 1: Figure S1). Although previous reports had evaluated the association between the lung or heart dosimetric parameters and treatment-related toxicities in esophageal cancer patients [19–25], the effects of lung and cardiac radiation doses on survival outcomes remain unknown.

We hypothesized that the radiation dose to the lungs or heart could affect survival outcomes in esophageal cancer patients. This study aimed to determine the associations between lung or heart radiation dose-volumes and the prognosis of esophageal cancer after trimodal therapy.

## Methods

### Patients

Patients with esophageal cancer who were treated with trimodal therapy between 2010 and 2015 at two tertiary centers were reviewed. The eligibility criteria were histologically proven esophageal cancer without clinical evidence of metastatic disease, Eastern Cooperative Oncology Group (ECOG) performance status ≤1, and full pretreatment evaluation data (i.e., history taking, physical examination, hematological and biochemical tests, upper gastrointestinal panendoscopy, computed tomography [CT], and whole-body F-18 fluorodeoxyglucose positron emission tomography/ computed tomography [18-FDG PET/CT]). Staging was based on the 7th edition of the Union for International Cancer Control/American Joint Committee on Cancer TNM classification system. Pretreatment feeding jejunostomy was performed after consulting with a nutritionist. Patients with a history of prior chemotherapy, radiotherapy, or any other cancer prior to esophageal cancer and those with synchronous double cancer were excluded. The study protocol was approved by the institutional review boards (IRB) of both centers.

### Neoadjuvant chemoradiotherapy and surgery

The NCRT comprised concurrent radiotherapy and chemotherapy with cisplatin and 5-fluorouracil. Chemotherapy consisted of two cycles of cisplatin and 5-fluorouracil (5-FU). The cisplatin (50–75 mg/m^2^) was administered intravenously on days 1 and 29 of radiotherapy, while 5-FU (600–800 mg/m^2^) was administered every 24 h as a continuous infusion for 4 days on the same day when cisplatin administered. Chemoradiotherapy was stopped if grade ≥ 3 treatment-related toxicity occurred.

Intensity-modulated radiotherapy was performed once daily for 5 days a week at a dose of 40.0–50.4 Gy in 23–30 fractions. The gross tumor volume (GTV) consisted of the primary tumor and lymphadenopathy based on clinical findings and staging images. The target volume included primary tumor and lymphadenopathy plus a 1-cm circumferential margin and a 3- to 4-cm longitudinal margin. Elective nodal irradiation was also included in the target volume based on the physician’s discretion. The normal tissue constraints were as follows: maximal dose of 45 Gy to the spinal cord, lung volume receiving 20 Gy or greater radiation dose (V20) ≤30%, and mean heart dose ≤30 Gy. A dose-volume histogram (DVH) parameter of V_x_ was defined as the percentage of the total organ volume receiving a radiation dose of x (Gy) or more.

Restaging survey for clinical response evaluation included upper gastrointestinal endoscopy with biopsy, chest CT, and 18-FDG PET/CT approximately 2 to 4 weeks after completion of NCRT. Surgery for all patients was performed 4 to 8 weeks after completion of the NCRT regimen. All patients underwent curative resection with the following approaches: transhiatal esophagectomy with abdominal lymphadenectomy, Ivor Lewis esophagectomy with abdominal and thoracic lymphadenectomy, and 3-field esophagectomy with abdominal and thoracic lymphadenectomy.

### Pathological analysis

Pathological analysis was performed under observation by a pathologist in each center. Histopathologic examination indicated whether a complete resection was performed with no tumor within 1 mm of the resection margins (R0) or whether a vital tumor was present at 1 mm or less from the resection margin (R1). Tumor response was graded using the College of American Pathologist Cancer Protocol for Esophageal Carcinoma [26]. Tumor regression grade (TRG) 0 (complete response) indicated no residual cancer cells. TRG 1 (moderate response) was defined as minimal residual cancer; TRG 2 (minimal response) as partial regression of the tumor, and TRG 3 (poor response) no definitive identified response.

### Evaluation of the adverse events of NCRT and severity in postoperative morbidity

Toxicities were evaluated and graded according to the Common Terminology Criteria for Adverse Events version 4.0. Radiation pneumonitis was diagnosed based on clinical and radiographic findings. The presence of radiographic pneumonitis was not attributable to other causes such as infection or tumor recurrence. All postoperative complications, including pulmonary, cardiac, chylothorax, and anastomotic leakage complications, were recorded up to 30 days postoperatively or during the same hospital stay after surgery. Pulmonary complications included pneumonia, serious atelectasis, pneumothorax, pleural effusion, pulmonary embolus, and acute respiratory failure. Cardiac complications included dysrhythmia, myocardial infarction, and left ventricular failure. Other recorded data included the median length of hospital stay and death within 7, 30, and 60 days of surgery.

### Surveillance and recurrence evaluation

The patients were followed up every 3 months for the first year and then every 6 months thereafter. The follow-up evaluation included clinical examination, blood tests, chest/abdominal CT, and upper gastrointestinal panendoscopy with biopsies. Further imaging studies were performed if there were clinical suspicion for recurrence. Recurrence was diagnosed based on physical or radiographic examinations or pathological confirmation.

### Statistical analyses

Continuous data are presented as the mean ± standard deviation or median and interquartile range (IQR), as applicable, while categorical data are presented as numbers and percentages. Spearman’s correlation coefficient was used to assess relationships between dosimetric factors. Logistic regression models were used to test for associations between dosimetric factors and postoperative complications.

Survival was measured from the date of diagnosis to the date of events or last follow-up. The Kaplan-Meier method and log-rank test were used to estimate overall survival (OS) and progression-free survival (PFS). The log-rank test was used to evaluate inter-group survival differences. The association of clinical or dosimetric factors with survival outcomes was calculated by using Cox proportional hazards model. Variables with a *p* value < 0.1 in univariate analysis were selected for multivariate analysis. The receiver-operating characteristic (ROC) curves and Youden index were used to generate cut-off values for DVH parameters that were found to be significantly associated with survival outcomes in multivariate analysis. The area under the ROC curves (AUC) was also assessed to evaluate the discriminative power of ROC analysis. We evaluated survival outcome stratification according to the radiation dose constraints. All statistical analyses were performed using Statistical Package for the Social Sciences for Windows, SPSS® software v. 21.0 (IBM Corp., New York, NY; formerly SPSS Inc., Chicago, IL), and a *p* < 0.05 was considered statistically significant.

## Results

### Patient and treatment characteristics

The patient demographics and tumor and radiation dosimetry characteristics of the 123 patients are shown in Table 1. The mean age was 54.3 ± 7.6 years. The majorities of patients in this study were male, squamous cell carcinoma, clinical stage III disease, and current smokers. The correlations between dosimetric factors are shown in Additional file 2: Table S1. Lung V5 was moderately correlated with GTV and radiation dose (Spearman’s ρ for GTV, 0.62; *p* < 0.001; ρ for radiation dose, 0.54; *p* < 0.001). Lung V20 was weakly correlated with GTV and radiation dose.

**Table 1:** Patient and tumor characteristics

Characteristics	Overall (*n* = 123)
Age (years), mean ± SD	54.3 ± 7.6
Sex, n (%)	
Man	117 (95.1)
Woman	6 (4.9)
ECOG performance status, n (%)	
0	105 (85.4)
1	18 (14.6)
Pathology, n (%)	
SCC	120 (97.6)
Adenocarcinoma	3 (2.4)
Location, n (%)	
Upper	24 (19.5)
Middle	59 (48.0)
Lower	40 (32.5)
BMI (kg/m^2^), mean ± SD	22.1 ± 3.3
Histologic grade, n (%)	
Grade 1	12 (9.8)
Grade 2	88 (71.5)
Grade 3	23 (18.7)
Smoking, n (%)	
No (never smoked or quitted)	7 (5.7)
Yes (current smoker)	116 (94.3)
Clinical T stage, n (%)	
cT1	1 (0.8)
cT2	36 (29.3)
cT3	80 (65.0)
cT4a	6 (4.9)
Clinical N stage, n (%)	
cN0	14 (11.4)
cN1	57 (46.3)
cN2	39 (31.7)
cN3	13 (10.6)
cTNM stage, n (%)	
II	31 (25.2)
III	92 (74.8)
Gross tumor volume (ml), mean ± SD	100.9 ± 73.2
Radiation dose (Gy), median (IQR)	44.0 (43.2–45.0)
40–44 Gy, n(%)	62 (50.4)
45–50.4 Gy, n(%)	61 (49.6)
Target volume (ml), mean ± SD	527.6 ± 228.5
Dose-volume of lung^a^ (%), mean ± SD	
V5	74.0 ± 21.2
V20	20.8 ± 6.4
Mean heart dose (Gy), mean ± SD	18.3 ± 7.6

*Abbreviations*: *BMI* body mass index, *ECOG* Eastern Cooperative Oncology Group, *IQR* interquartile range, *SCC* squamous cell carcinoma, *SD* standard deviation

^a^Vx = volume (mL) of lung receiving X Gy or more

The median duration of NCRT was 36 (IQR: 31–40) days. All patients completed the planned radiotherapy regimen. A total of 123 (100%) and 116 (94.3%) patients completed the first and second courses of chemotherapy, respectively. During NCRT, most cases of acute toxicity of grade ≥ 3 were those of hematological toxicity (*n* = 54, 43.9%), followed by weight loss (*n* = 21, 17.1%). Four patients (3.2%) experienced symptomatic (grade 2) pneumonitis, and none had grade ≥ 3 pneumonitis. One patient (0.8%) experienced severe esophagitis (Table 2).

**Table 2:** Adverse events during neoadjuvant chemoradiotherapy and after surgery

Event	Any grade, n (%)	Grade ≥ 3, n (%)
Postoperative events		
Pulmonary complications^a^	36 (27.6%)	15 (12.2)
Cardiac complications^c^	21 (17.1%)	7 (5.7)
Chylothorax	15 (12.2)	2 (1.6)
Anastomotic leakage	21 (17.1)	9 (7.3)
Postoperative mortality^b^		
7 days	0 (0)	–
30 days	3 (2.4%)	–
60 days	1 (0.8%)	–
Events during chemoradiotherapy		
Hematological toxicity^d^	113 (91.9)	54 (43.9)
Weight loss	101 (82.1)	21 (17.1)
Esophagitis	114 (92.7)	1 (0.8)
Radiation pneumonitis	56 (45.5)	0 (0)
Fatigue	77 (62.6)	0 (0)

^a^Pulmonary complications included pneumonia, serious atelectasis, pneumothorax, pleural effusion, pulmonary embolus, and acute respiratory failure

^b^The causes of postoperative mortality for the 4 patients were all postoperative complication of sepsis

^c^Cardiac complications included dysrhythmia, myocardial infarction, and left ventricular failure. All of 7 patients had grade 3 cardiac dysrhythmia; no patients had grade ≥ 4 cardiac complications

^d^Hematological toxicity included leukopenia, neutropenia, and thrombocytopenia

The median interval between completion of NCRT and surgery was 6.7 (IQR: 5.4–8.3) weeks. Postoperative adverse events are also summarized in Table 2. Thirty-six (27.6%) patients had pulmonary complications of any grade; 15 (12.2%) had grade ≥ 3 pulmonary complications. A total of 7 (5.7%) patients had grade 3 cardiac dysrhythmia; one patient had grade 3 cardiac dysrhythmia during the same hospital stay after surgery, and 6 patients had grade 3 cardiac dysrhythmia during follow-up. No patients had grade ≥ 4 cardiac complications. The median interval from the date of surgery to diagnosis of grade 3 cardiac dysrhythmia was 3.1 months (range, 1.3–32.8 months). Median postoperative hospital stay was 21 days (IQR, 16–28 days). Lung V5 was associated with pulmonary complications (Table 3). Dosimetric factors were not significantly associated with grade ≥ 3 cardiac complication and 30-day and 60-day postoperative mortality. The postoperative hospital stay was not correlated with mean heart dose and lung V20 (Spearman’s ρ for mean heart dose, 0.01; *p* = 0.96; ρ for lung V20, 0.09; *p* = 0.35) and weakly correlated with lung V5 (Spearman’s ρ for mean heart dose, − 0.25; *p* = 0.01).

**Table 3:** Univariate logistic regression of dosimetric factors and postoperative adverse events

	Pulmonary complications^a^		Cardiac complications^b^		30-day mortality		60-day mortality	
Characteristics	OR (95% CI)	*p*-value	OR (95% CI)	*p*-value	OR (95% CI)	*p*-value	OR (95% CI)	*p*-value
Gross tumor volume	1.00 (0.99–1.01)	0.17	1.00 (0.99–1.00)	0.67	1.00 (0.98–1.02)	0.92	1.01 (1.00–1.02)	0.09
Target volume	1.00 (1.00–1.00)	0.20	1.00 (0.99–1.00)	0.39	1.00 (0.98–1.01)	0.24	1.00 (0.99–1.01)	0.92
Radiation dose	1.00 (1.00–1.00)	0.83	1.00 (1.00–1.00)	0.61	1.00 (1.00–1.00)	0.90	1.00 (1.00–1.00)	0.88
DVH of lung^c^								
V5	1.02 (1.00–1.04)	0.03	0.98 (0.95–1.02)	0.32	1.04 (0.97–1.12)	0.32	1.04 (0.98–1.11)	0.22
V20	0.99 (0.93–1.05)	0.72	0.94 (0.82–1.07)	0.35	1.00 (0.79–1.16)	0.65	0.99 (0.85–1.16)	0.91
Mean heart dose	1.00 (1.00–1.00)	0.82	0.99 (1.00–1.00)	0.13	1.00 (1.00–1.00)	0.71	1.00 (1.00–1.00)	0.31

*Abbreviations*: *CI* confidence interval, *DVH* dose-volume histogram, *GTV* gross tumor volume, *OR* odds ratio

^a^Any grade

^b^Grade ≥ 3 complications. The 7 patients had grade 3 cardiac dysrhythmia were analyzed

^c^Vx = volume (mL) of lung receiving X Gy or more

Table 4 shows the pathological staging and effects of NCRT. Overall, R0 resections after NCRT were achieved in 104 (84.6%) patients; the R0 resection rate according to tumor locations was 75% for upper third tumors, 81.7% for middle third tumors, and 94.9% for lower third tumors (*p* = 0.051). The R0 resection rates were 89.2 and 70.0% for patients with pathological stage 0-II and III, respectively (*p* = 0.02). A total of 46 (37.4%) patients achieved TRG 0 (pathological complete response), and of 11 (8.9%) patients had TRG 1 (minimal residual cancer).

**Table 4:** Surgery and pathological staging for patients

Characteristics	Overall (*n* = 123)
Time from NCRT to surgery (weeks), median (IQR)	6.7 (5.4–8.3)
Pathological T stage, n (%)	
pT0	52 (42.3)
pT1	15 (12.2)
pT2	19 (15.4)
pT3	35 (28.5)
pT4a	2 (1.6)
Pathological N stage, n (%)	
pN0	82 (66.7)
pN1	24 (19.5)
pN2	13 (10.6)
pN3	4 (3.3)
Pathological TNM stage, n (%)	
pT0N0	46 (37.4)
I	14 (11.4)
II	33 (26.8)
III	30 (24.4)
Tumor regression grade, n (%)	
0	46 (37.4)
1	11 (8.9)
2	60 (48.8)
3	6 (4.9)
R0 resection, n (%)	
All location	104 (84.6)
Upper	18 (14.6)^a^
Middle	48 (39.0)^a^
Lower	38 (30.9)^a^

*Abbreviations*: *NCRT* neoadjuvant chemoradiotherapy, *IQR* interquartile range

^a^Location specific R0 resection rate: upper (75.0%), middle (81.7%), lower (94.9%)

### Survival outcome and cause of death

The median follow-up was 28.3 months (range: 4.7–92.8 months). By the last follow-up, 65 patients (52.8%) had died: 54 (83.1%) from esophageal cancer, 7 (10.8%) from other medical diseases, and 4 (6.2%) from postoperative complication of sepsis (2 patients died due to bacterial pneumonia [both with a lung V20 of 24%] and others due to intra-abdominal infectious diseases [lung V20 of 17 and 15%]).

**Fig. 1:**
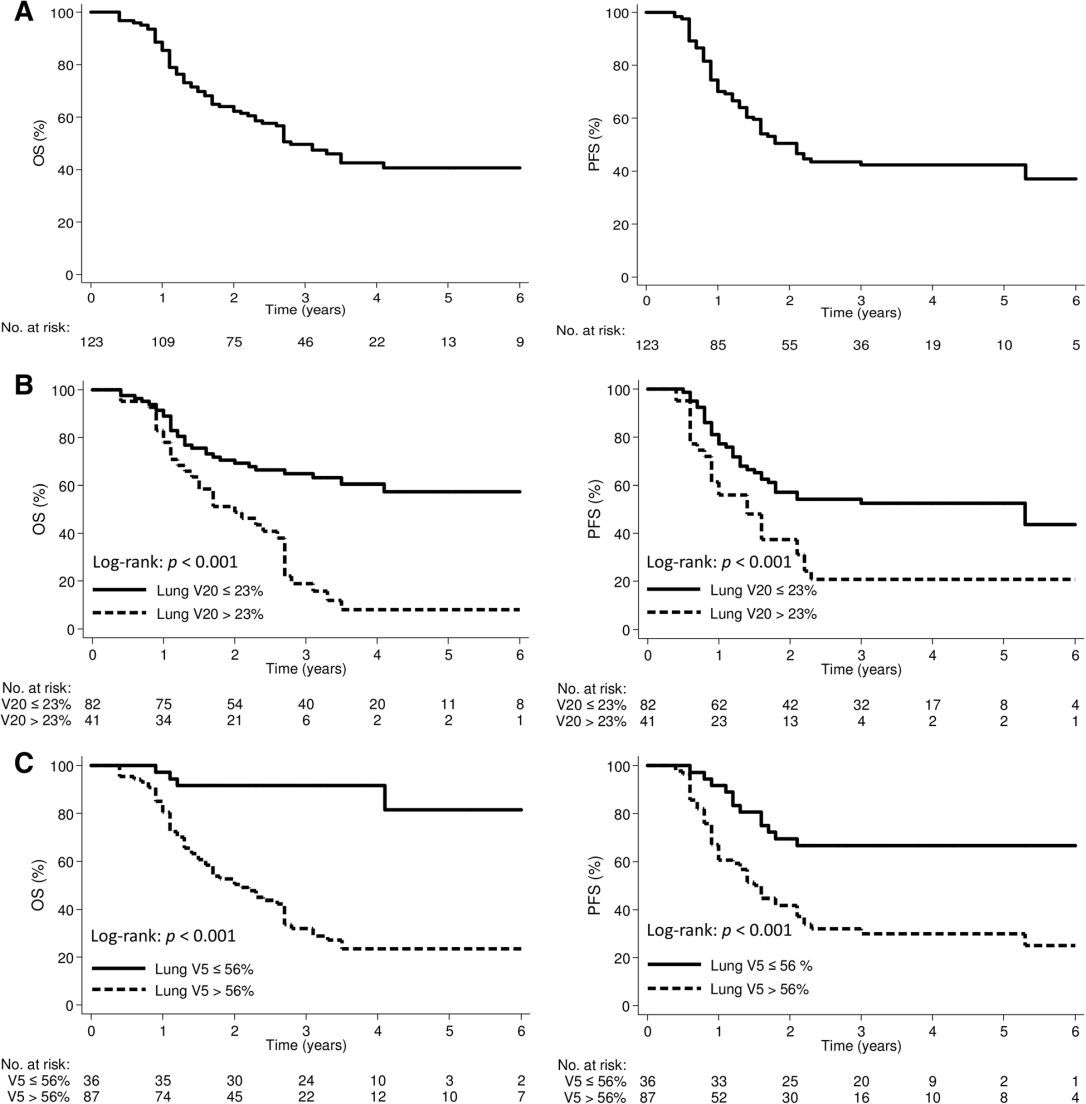
Kaplan-Meier curve demonstrating overall survival and progression-free survival according to **a** all patients, **b** lung V20, and **c** lung V5 groups. V_x_ was defined as the percentage of the total organ volume receiving a radiation dose of x (Gy) or more

The median OS and PFS were 34.0 months (95% CI, 27.4–40.6 months) and 24.8 months (95% CI, 18.9–30.7 months), respectively (Fig. 1a). Table 5 shows the results of the univariate and multivariate analyses. Multivariate analysis showed that R1 resection (hazard ratio [HR], 3.63; 95% confidence interval [CI]: 1.59–8.28, *p* = 0.002), TRG 2/3 (HR, 2.84; 95% CI: 1.50–5.37, *p* = 0.001), lung V20 (HR, 1.09; 95% CI: 1.04–1.14; *p* < 0.001) and lung V5 (HR, 1.02; 95% CI: 1.00–1.05; *p* = 0.03) were associated with OS. The R1 resection (HR, 4.16; 95% CI: 2.11–8.19, *p* < 0.001), TRG 2/3 (HR, 2.31; 95% CI: 1.30–4.12, *p* = 0.004), and lung V20 (HR, 1.07; 95% CI: 1.03–1.12; *p* = 0.001) were associated with PFS.

**Table 5:** Univariate and multivariate Cox proportional hazards model for overall survival and progression-free survival

Characteristics	OS				PFS			
	Univariate		Multivariate		Univariate		Multivariate	
	HR (95% CI)	*p*-value	HR (95% CI)	*p*-value	HR (95% CI)	*p*-value	HR (95% CI)	*p*-value
Age	0.97 (0.94–1.00)	0.08	0.97 (0.93–1.00)	0.08	0.96 (0.93–1.00)	0.03	0.97 (0.93–1.00)	0.07
BMI	0.99 (0.91–1.07)	0.74			1.05 (0.97–1.13)	0.22		
ECOG PS (1 vs. 0)	1.56 (0.83–2.91)	0.17			1.39 (0.74–2.61)	0.30		
Clinical T (T3–4 vs. T1–2)	1.06 (0.62–1.80)	0.84			0.93 (0.54–1.58)	0.78		
Clinical N (N+ vs. N0)	1.16 (0.50–2.70)	0.72			1.79 (0.72–4.46)	0.21		
cTNM stage (III vs. II)	0.89 (0.50–1.56)	0.67			0.68 (0.40–1.17)	0.16		
Time from NCRT to surgery	1.01 (0.94–1.09)	0.72			0.98 (0.91–1.06)	0.59		
Surgical margin (R1 vs. R0)	2.62 (1.41–4.85)	0.002	3.63 (1.59–8.28)	**0.002**	4.37 (2.46–7.77)	< 0.001	4.16 (2.11–8.19)	**< 0.001**
TRG (2/3 vs. 0/1)	3.42 (2.00–5.84)	**< 0.001**	2.84 (1.50–5.37)	**0.001**	2.39 (1.44–3.98)	0.001	2.31 (1.30–4.12)	**0.004**
Gross tumor volume	1.003 (1.000–1.005)	0.09	0.998 (0.994–1.002)	0.27	1.001 (0.998–1.004)	0.44		
Target volume	1.00 (1.00–1.00)	0.12			1.00 (1.00–1.00)	0.63		
Radiation dose	1.00 (1.00–1.00)	0.16			1.00 (1.00–1.00)	0.32		
DVH of lung^a^								
V5	1.02 (1.01–1.03)	0.001	1.02 (1.00–1.05)	**0.03**	1.01 (1.00–1.02)	0.03	1.01 (0.99–1.03)	0.21
V20	1.07 (1.04–1.11)	< 0.001	1.09 (1.04–1.14)	**< 0.001**	1.06 (1.02–1.10)	0.002	1.07 (1.03–1.12)	**0.001**
Mean heart dose	1.00 (1.00–1.01)	0.03	1.00 (1.00–1.00)	0.55	1.00 (1.00–1.00)	0.41		

*Abbreviations*: *BMI* body mass index, *CI* confidence interval, *DVH* dose-volume histogram, *ECOG* Eastern Cooperative Oncology Group, *HR* hazard ratio, *NCRT* neoadjuvant chemo-radiotherapy, *TRG* tumor regression grade, *PS* performance status

Bolded *p*-values are those significant with a *p* < 0.05

^a^Vx = volume (mL) of lung receiving X Gy or more

### Receiver-operating characteristic curve analysis for survival outcome

The ROC analysis for lung V20 and V5 revealed that the optimal cut-off points were 23% (AUC: 0.72; 95% CI: 0.63–0.81; *p* < 0.001) and 56% (AUC: 0.68; 95% CI: 0.57–0.78; *p* = 0.001), respectively. The OS and PFS curves according to the lung dose cut-offs are shown in Fig. 1. The OS and PFS were significantly lower in patients with lung V20 > 23% or V5 > 56%. The 5-year OS and PFS for patients with lung V20 ≤ 23% vs. > 23% were 54.4% vs. 5% (*p* < 0.001) and 50.1% vs. 20.0% (*p* = 0.004) respectively. For lung V5 cutoff value of ≤56 and > 56%, the 5-year OS and PFS were 81.5% vs. 23.4% (*p* < 0.001) and 66.7% vs. 29.7% (*p* < 0.001), respectively.

**Fig. 2:**
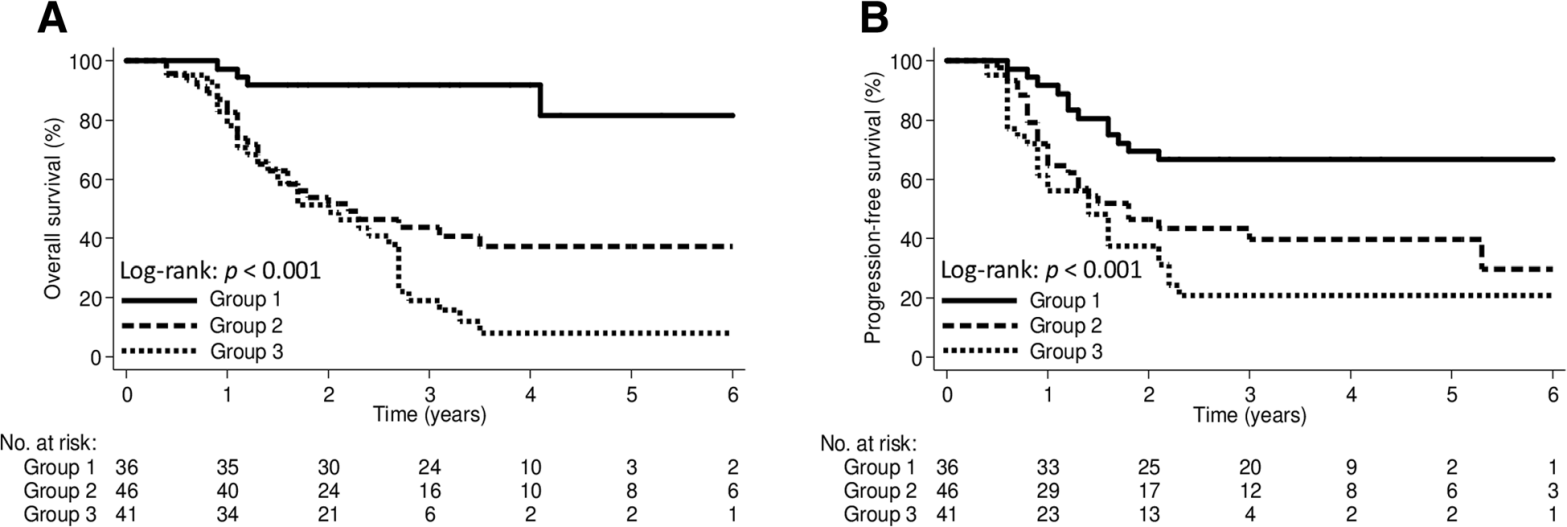
The Kaplan-Meier curve demonstrating **a** overall survival and **b** progression-free survival according to lung V20 and V5 groups. Group 1, V20 ≤ 23% and V5 ≤ 56%; Group 2, V20 ≤ 23% or V5 ≤ 56%; Group 3, V20 > 23% and V5 > 56%. V_x_ was defined as the percentage of total organ volume receiving a radiation dose of x (Gy) or more

We categorized the patients into 3 groups according to lung V20 and V5 values to further evaluate the effect of lung dose on outcomes. Group 1 included patients with both lung V20 ≤ 23% and V5 ≤ 56%; group 2 included patients with either lung V20 ≤ 23% or V5 ≤ 56%; group 3 included patients with lung V20 > 23% and V5 > 56%. The 5-year OS rates in group 1, group 2, and group 3 were 81.5, 37.0, and 7.9%, respectively (*p* < 0.001; Fig. 2a); the corresponding PFS rates were 66.7, 39.2, and 20.5%, respectively (*p* < 0.001; Fig. 2b).

## Discussion

The present study evaluated the effect of radiation doses to the lung and heart on survival outcomes in esophageal cancer patients receiving trimodal therapy. We found that higher lung radiation dose-volume was associated with worse survival outcomes, and the cutoff values of V20 and V5 to prevent mortality were ≤ 23% and ≤ 56%, respectively. The mean heart dose was not associated with survival.

Constraining the lung dose may minimize the risk of postoperative pulmonary complications and improve survival outcomes in patients with esophageal cancer receiving trimodal therapy. Previous studies have evaluated the association between lung dose and radiation pneumonitis and postoperative pulmonary complications in patients undergoing radiotherapy for esophageal cancer [19–25], while the prognostic significance of lung dose was unclear. Ou et al. reported that lung radiation dose was associated with OS in esophageal cancer patients receiving definitive or neoadjuvant chemoradiotherapy [27]. They suggested that esophageal cancer outcomes may be improved by minimizing lung dose, particularly the volume receiving 20 Gy or more. In the present study, we found that not only lung V20, but also the volume receiving a low radiation dose (5 Gy), was associated with survival outcomes. We also found lung V5 was associated with postoperative pulmonary complications. However, the effect of meeting only one lung constraint (either V20 or V5) on survival outcomes is unknown. We found that patients with either or both lung V20 > 23% and V5 > 56% had poorer survival outcomes than patients with both lung V20 ≤ 23% and V5 ≤ 56%.

A larger GTV is a predictor of poorer survival outcome in esophageal cancer [28, 29]. A larger GTV might also contribute to a larger target volume, thereby increasing the lung radiation dose-volume. In this study, the lung V5 was moderately correlated with GTV; while V20 was weakly correlated with GTV. A possible explanation is that we mainly constrained lung V20 in this study and V5 was an alternative lung dose-volume constraint. In addition, we found GTV was not associated with survival outcomes. These findings suggest that the lung volume receiving low radiation doses should be minimized to improve outcomes for esophageal cancer patients. The use of novel radiotherapy techniques or modalities such as proton beam therapy might optimize outcomes in esophageal cancer patients [30–35].

The effects of lung volume receiving low radiation doses on outcomes are not well-understood. In the present study, lung V5 was associated with postoperative pulmonary complications and OS. Fractionated low-dose radiation may increase DNA damage and affect replication, and induce apoptosis in the lung parenchyma [36]. Furthermore, the lungs serve as sites of platelet biogenesis and act as a reservoir for resident megakaryocytes and hematopoietic progenitor cells. The megakaryocytes are a rich source of cytokines and growth factors that play a role in the pathogenesis of inflammatory or fibrotic lung diseases [37]. It has been hypothesized that exposure of a larger lung volume to low radiation doses may influence the host immunity and microenvironment and affect survival outcomes. The lymphocyte nadir during NCRT was found to be associated with survival and treatment response in esophageal cancer [38, 39]. Irradiation might induce interleukin-6 and lead to activation of JAK/STAT3 signaling in both tumor cells and tumor-infiltrating immune cells, which can promote tumor cell proliferation, survival, invasiveness, and metastasis [40–42]. The treatment strategy targeting components of the IL-6/JAK/STAT3 signaling pathway might play a role in optimizing treatment outcomes in esophageal cancer. A future study evaluating the effects of low-dose radiation to the lung on the lung microenvironment, cytokines, and treatment outcomes is needed.

Recently, a higher heart dose was reported to be associated with worse OS and higher risk of cardiac events in lung cancer [12–15]. In the present study, 21 (17.1%) patients had postoperative cardiac complications of any grade. Among the 7 patients who had grade 3 cardiac dysrhythmia, 6 were found during follow-up, with a median time to occurrence of 3.1 months (range, 1.3–32.8 months) after surgery. No patients died of cardiac complications. Furthermore, grade 3 cardiac dysrhythmia was not associated with mean heart dose. Our study also showed no association between mean heart dose and survival outcomes in esophageal cancer, which is consistent with the findings of recent studies [43, 44]. A possible explanation is that the prescribed radiation dose in NCRT was lower for esophageal cancer (40–50 Gy) than for lung cancer (60–70 Gy). In addition, the major cause of death in this study was cancer-related death, and radiation-induced cardiac events or cardiac death might not have been detected. The latency period between radiotherapy and the associated clinical cardiac events can range from years to decades [14, 15, 45, 46]. Further analysis of cardiac dose and cardiac events, in addition to survival outcomes, is needed to avoid underestimation of cardiac toxicity and to provide appropriate dose constraints for the heart in esophageal cancer.

Previous studies revealed that major pathological response (complete or near complete response) after NCRT or R0 resection was associated with favorable survival outcome in esophageal cancer patients [47–49]. The present study also found the TRG 0/1 and R0 resections were independently associated with survival outcomes. The pathological complete response rate (37.4%) in our study was comparable with other reports on this subject [6–8, 47, 48]. However, the overall R0 resection rate of 84.6% was relative lower compared to that of previous studies [6, 8, 49]. Possible reasons include that, in this study, 19.5 and 74.8% of patients exhibited upper location and clinical stage III disease, respectively. In addition, 24.4% of patients had pathological stage III disease. One large multicenter European study revealed that independent factors significantly associated with an R1 resection margin included an upper third esophageal tumor location and pathological stage III [49]. We also found the R0 resection rates were lower in patients with upper tumor location or pathological stage III. Although this study had a lower overall R0 resection rate compared to that of previous studies [6, 8, 49], this real-world outcome research highlighted the prognostic role of major pathological response and R0 resection.

The optimal radiation dose of NCRT for esophageal cancer remains controversial, and previous retrospective studies have revealed wide variation in the radiation dose [50–53]. Buckstein et al. reported neoadjuvant radiation dose for esophageal cancer was not associated with differences in OS [50]. The use of a 41.4 Gy dose is increasing, and several studies also reported 41.4 Gy was associated with reduced perioperative mortality and increased rates of esophagectomy without negatively impacting OS, R0 resection, or complete pathologic response [50–52]. Semenkovich et al. reported a 50.4 Gy dose was associated with a higher likelihood of pathological complete response without adversely affecting perioperative mortality compared with that of a 45 Gy dose [53]. In the present study, the radiation doses ranged from 40 to 50.4 Gy, and we also found radiation dose was not associated with OS. In addition, it should be noted that the chemotherapy regimen in this study was mainly cisplatin and 5-fluorouracil rather than carboplatin and paclitaxel in the CROSS regimen. However, the difference in OS between these two chemotherapy regimens may not be significant [10]. Given the retrospective nature of the studies mentioned above, prospective trials are needed to evaluate the optimal dose of NCRT.

There were several limitations to our study. First, this was a retrospective study with a small number of patients and a short follow-up duration. Second, the cytokine profiles were not available in this retrospective study; therefore, the associations of radiation dose to the lung and cytokines with survival outcomes could not be assessed. Third, the majority of our patients had squamous cell histology, so our results may not be fully applicable to patients with other histologic types. Despite these limitations, the quality of care with regard to NCRT and surgery in this study was consistent with the current standards of practice, and our results were comparable with those of previous studies [6–8].

In conclusion, the present study showed that lung radiation dose-volume was associated with survival outcome in esophageal cancer patients who underwent NCRT and surgery, suggesting that esophageal cancer outcomes may be improved by minimizing the lung dose. In addition, the lung volume receiving low radiation doses may also affect survival outcomes and should also be minimized.

## Additional files

Additional file 1: **Figure. S1.** Figure showing the isodose line of 20 Gy (outlined in yellow) and 5 Gy (outlined in blue) in radiotherapy planning. Esophageal cancer is outlined in green and the prescribed dose is 48 Gy. (TIF 1748 kb)

Additional file 2: **Table S1.** Spearman’s ρ correlations and scatter map between dosimetric parameters. (DOCX 561 kb)

## Abbreviations

18-FDG: F-18 fluorodeoxyglucose
5-FU: 5-fluorouracil
AUC: Area under the curve
CI: Confidence interval
CT: Computed tomography
DNA: Deoxyribonucleic acid
DVH: Dose-volume histogram
ECOG: Eastern Cooperative Oncology Group
HR: Hazard ratio
IQR: Interquartile range
IRB: Institutional review boards
NCRT: Neoadjuvant chemoradiotherapy
OS: Overall survival
PET/CT: Positron emission tomography–computed tomography
PFS: Progression-free survival
ROC: Receiver-operating characteristic
