# Supplementary figures and images for: Prognostic significance of lung radiation dose in patients with esophageal cancer treated with neoadjuvant chemoradiotherapy
Source: Radiat Oncol. 2019 May 24;14:85. doi: 10.1186/s13014-019-1283-3 (PMC6534831; doi:10.1186/s13014-019-1283-3)

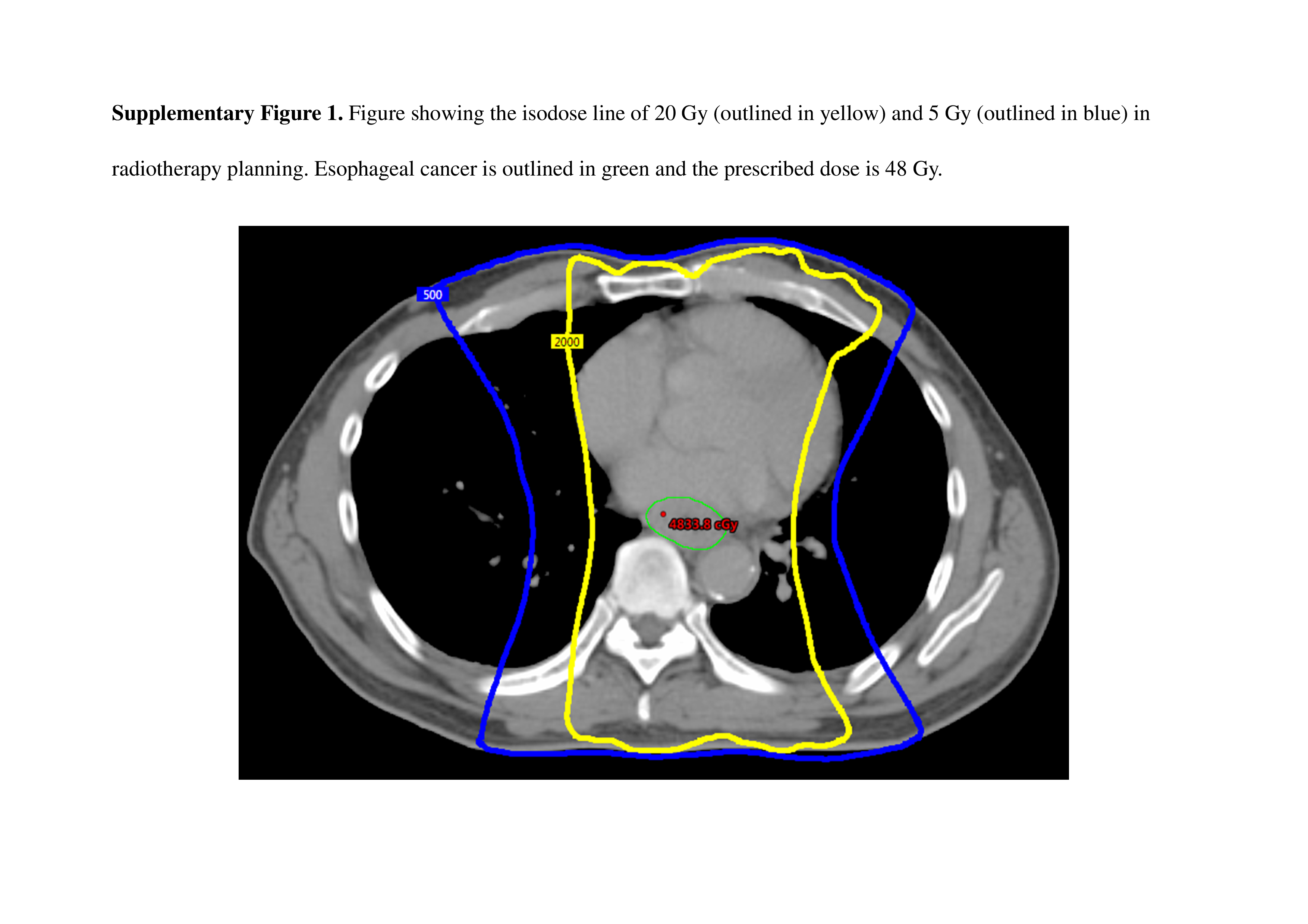

Supplement: Supplementary file 1 — Figure. S1. Figure showing the isodose line of 20 Gy (outlined in yellow) and 5 Gy (outlined in blue) in radiotherapy planning. Esophageal cancer is outlined in green and the prescribed dose is 48 Gy. (TIF 1748 kb) [file 13014_2019_1283_MOESM1_ESM.tif]
