# Supplementary material for: Prognostic significance of lung radiation dose in patients with esophageal cancer treated with neoadjuvant chemoradiotherapy
Source: Radiat Oncol. 2019 May 24;14:85. doi: 10.1186/s13014-019-1283-3 (PMC6534831; doi:10.1186/s13014-019-1283-3)
Supplement: Supplementary file 2 — Table S1. Spearman’s ρ correlations and scatter map between dosimetric parameters. (DOCX 561 kb) [file 13014_2019_1283_MOESM2_ESM.docx]

**Supplementary Table S1** Spearman’s ρ correlations and scatter map between dosimetric parameters

| Spearman’s ρ *p*-value | Mena heart dose | Lung V5* | Lung V20* | GTV | Target volume | Radiation dose |
| --- | --- | --- | --- | --- | --- | --- |
| Heart mean dose | 1 | 0.33  <0.001 | 0.36  <0.001 | 0.38  <0.001 | 0.17  0.06 | 0.17  0.06 |
| Lung V5* | 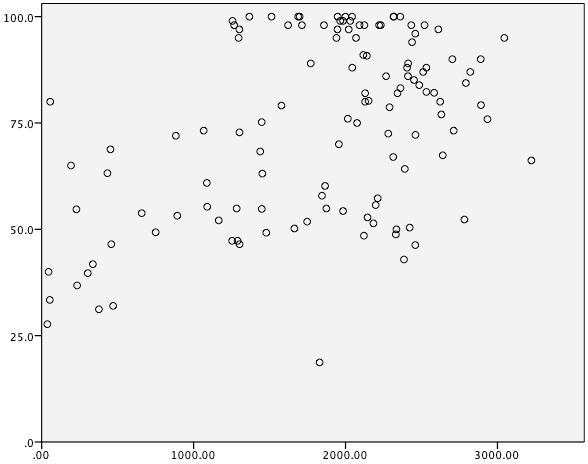 | 1 | 0.15  0.10 | **0.62** †  **<0.001** | 0.12  0.04 | **0.54** †  **<0.001** |
| Lung V20* | 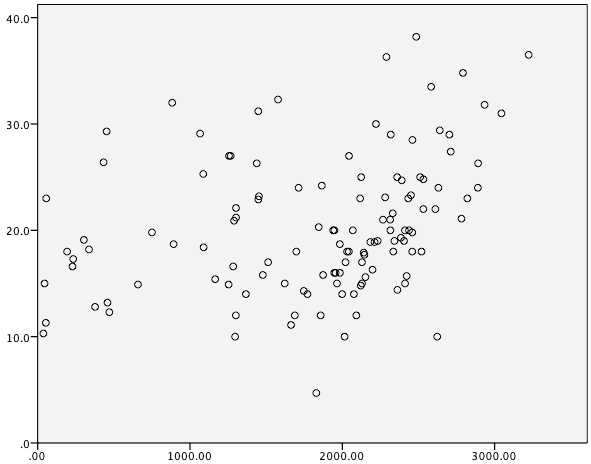 | 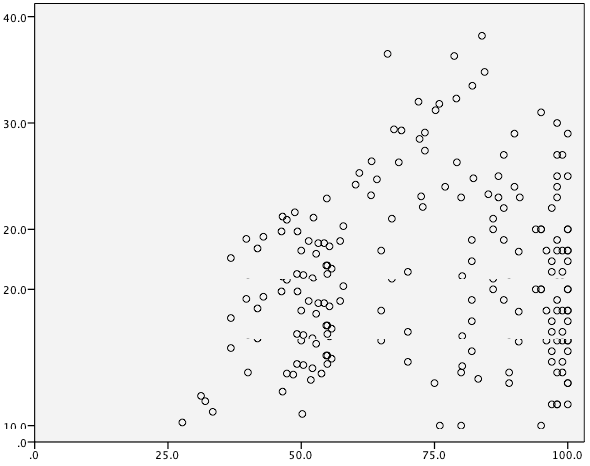 | 1 | 0.21  0.02 | 0.29  0.001 | 0.12  0.26 |
| GTV | 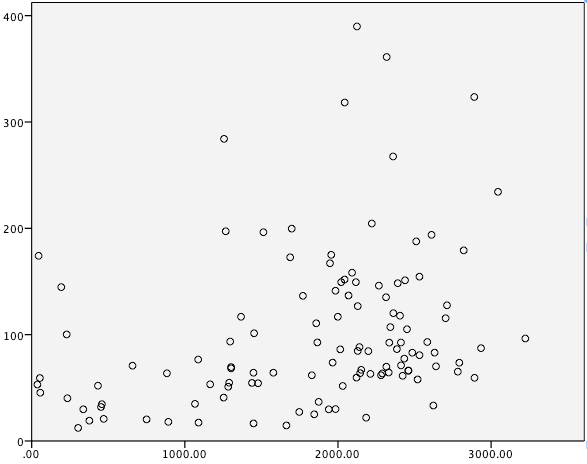 | 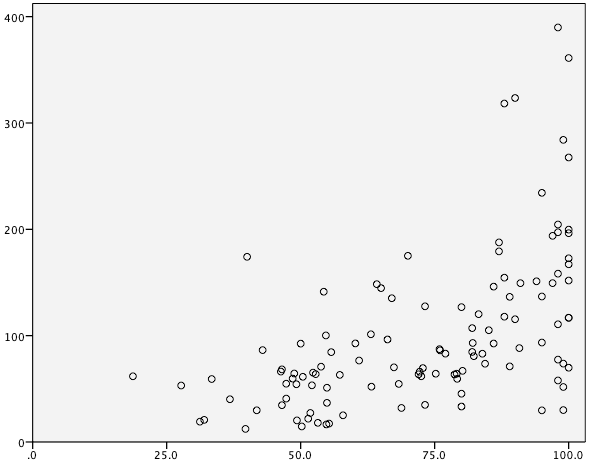 | 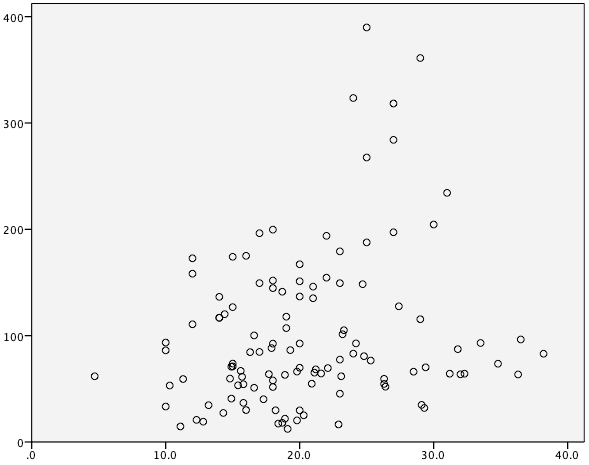 | 1 | 0.34  <0.001 | **0.46** †  **<0.001** |
| Target volume | 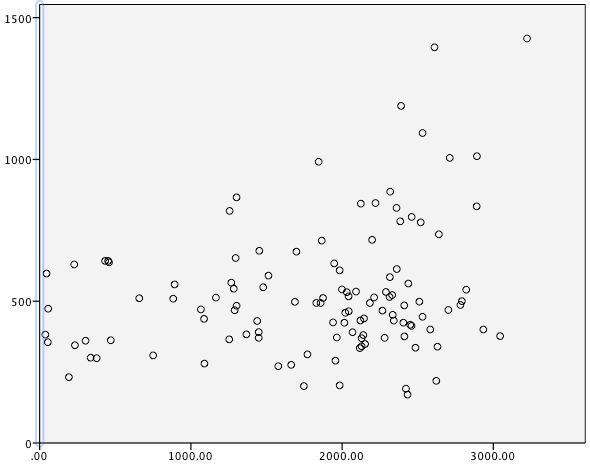 | 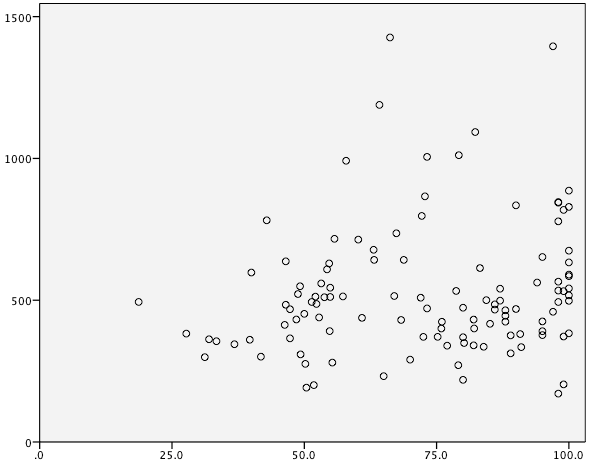 | 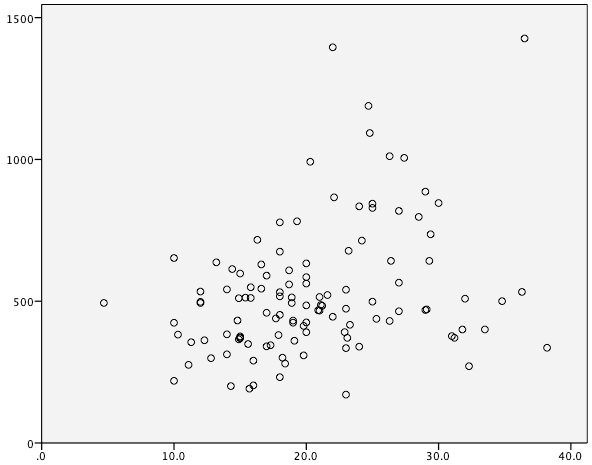 | 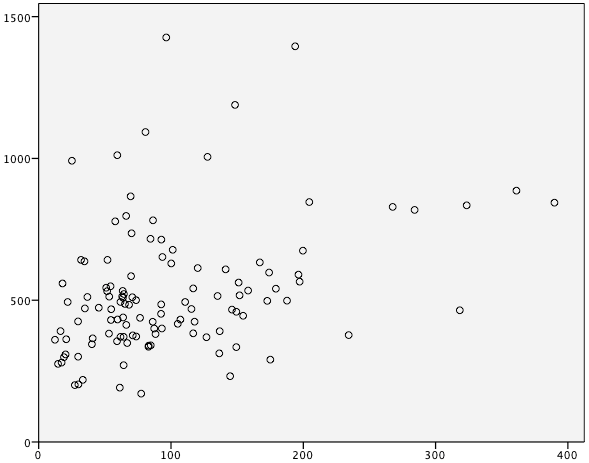 | 1 | 0.02  0.86 |
| Radiation dose | 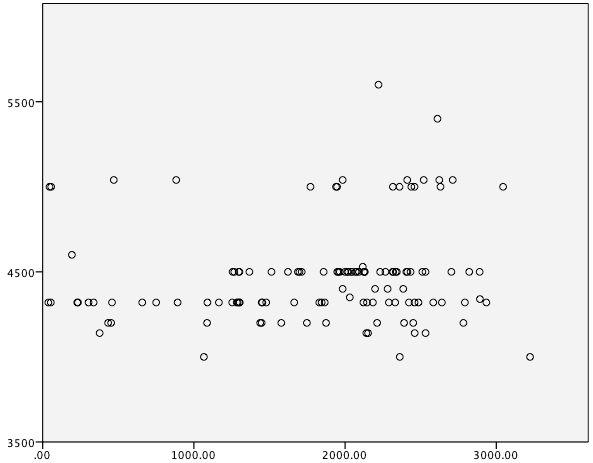 | 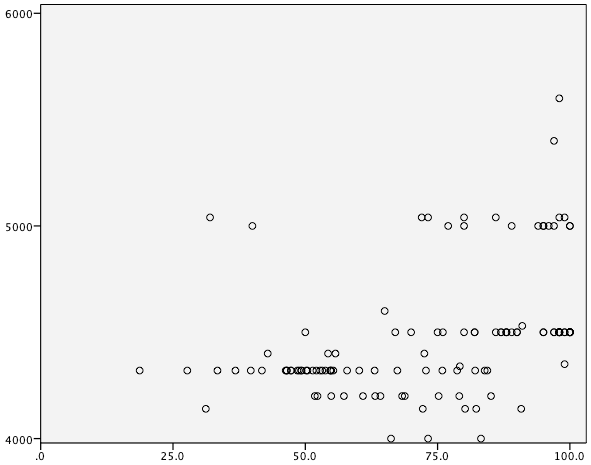 | 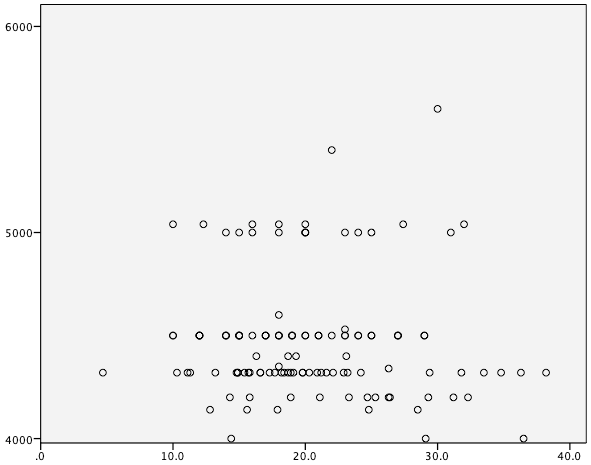 | 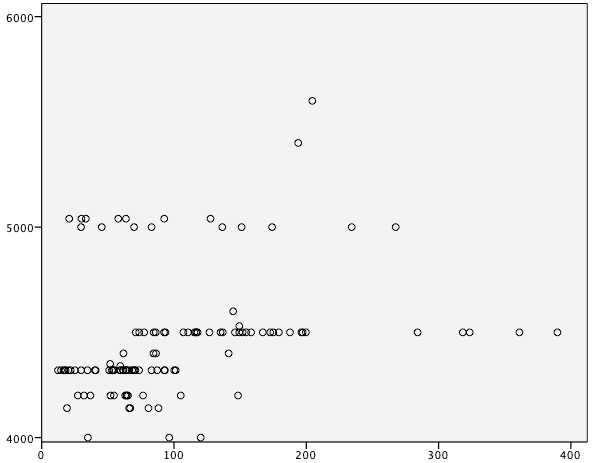 | 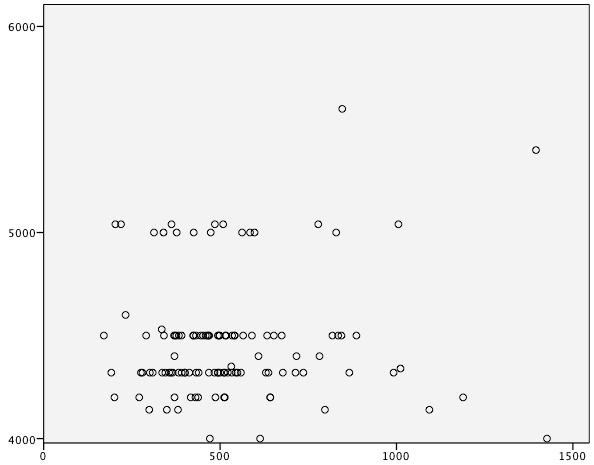 | 1 |
| Abbreviation: GTV, gross tumor volume.  * Vx = volume (mL) of lung receiving X Gy or more.  † Bold Spearman’s ρ and *p*-value indicated a moderate correlation between two variables (defined as Spearman’s ρ range 0.40 to 0.69) | | | | | | |
